# Free-Space to SMF Integration and Green to C-Band Conversion Based on PPLN

**DOI:** 10.3390/s24248162

**Published:** 2024-12-21

**Authors:** Takahiro Kodama, Kiichiro Kuwahara, Ayumu Kariya, Rikizo Ikuta

**Affiliations:** 1 Faculty of Engineering and Design, Kagawa University, 2217-20 Hayashi-cho, Takamatsu 761-0396, Kagawa, Japan; s21t416@kagawa-u.ac.jp (K.K.); s23g406@kagawa-u.ac.jp (A.K.); 2 Graduate School of Engineering Science, Osaka University, Toyonaka 560-8531, Osaka, Japan; ikuta.rikizo.es@osaka-u.ac.jp; 3 Center for Quantum Information and Quantum Biology, Osaka University, Toyonaka 560-0043, Osaka, Japan

**Keywords:** optical remote sensor, optical wavelength conversion, periodically poled lithium niobate

## Abstract

In this study, we experimentally demonstrate a PPLN-based free-space to SMF (single-mode fiber) conversion system capable of efficient long-wavelength down-conversion from 518 nm, optimized for minimal loss in highly turbid water, to 1540 nm, which is ideal for low-loss transmission in standard SMF. Leveraging the nonlinear optical properties of periodically poled lithium niobate (PPLN), we achieve a wavelength conversion efficiency of 1.6% through difference frequency generation while maintaining a received optical signal-to-noise ratio of 10.4 dB. Our findings underscore the potential of integrating PPLN-based wavelength conversion with fiber optic networks, offering a viable solution for next-generation optical sensor systems that demand real-time, low-latency, and reliable data transmission. This work represents a significant advancement in developing robust and efficient optical sensor technologies, addressing the challenges associated with long-distance transmission and broad-linewidth light sources in optical remote sensing applications.

## 1. Introduction

The increasing demand for high-precision, non-contact, and remote gas sensing applications has underscored the necessity for advanced optical sensor technologies [1,2,3,4,5,6,7]. Optical sensors offer distinct advantages due to their specific interaction with various gas molecules such as ozone (O_3_), nitrogen dioxide (NO_2_), and certain volatile organic compounds (VOCs) [8,9,10]. However, the efficient transmission of green light sensor signals over long distances remains a significant hurdle, primarily due to transmission losses and the need for seamless integration with existing optical fiber networks. This challenge calls for further research and innovation in the field.

Underwater optical sensors have garnered attention for their ability to perform high-precision sensing along the longitudinal direction within water. These sensors are particularly effective for detecting gasses and oils underwater, where RF signals are significantly absorbed and scattered by water. Green light, especially around 518 nm, is suitable for underwater applications due to its relatively low absorption in water. Green light optical sensors are indispensable for detecting various underwater substances, such as methane (CH_4_), hydrogen sulfide (H_2_S), dissolved oxygen (O_2_), and oil hydrocarbons [11,12].

Despite these advantages, green light optical sensors with optical fibers face significant transmission losses compared to C-band optical sensor systems with optical fibers [13,14,15,16]. Transmission losses in the optical fibers become more pronounced as the transmission distance increases, requiring advanced system design. Integrating green light optical sensors with fiber optic technology is essential to mitigate these transmission losses and enhance overall performance. This integration can improve the range and reliability of green light optical sensor systems, particularly in applications requiring seamless transition between wireless and fiber hybrid optical sensors.

In addressing the latency and loss challenges in optical remote sensor systems, optical/optical (O/O) conversion types have emerged as a promising solution [17]. These free space-to-fiber converters (FSFCs) leverage all-optical wavelength conversion techniques, which are well suited for low-loss and low-latency applications because they can instantly process signals. The core of this technology involves the use of periodically poled lithium niobate (PPLN). This highly efficient nonlinear optical crystal not only facilitates various wavelength conversion processes [18], including second harmonic generation (SHG) [19], difference frequency generation (DFG) [20,21], sum frequency generation (SFG) [22,23], and optical parametric amplification (OPA) [24], but also holds immense potential for future advancements in optical sensor systems.

Recent advancements in short wavelength up-conversion using PPLN have shown promising results, such as converting a narrow-linewidth 1064 nm light source to a 532 nm signal through SHG [25]. There has been a report of wavelength conversion from 1550 nm to 516 nm using cascaded PPLN for sequential SHG and SFG [26]. These developments underscore the potential of PPLN in achieving high-efficiency wavelength conversion. However, the conversion process using PPLN is not without its challenges. It is susceptible to the linewidth of the incident light. The linewidth much broader than the acceptance bandwidth of several tens of GHz or more in cases of typical PPLN waveguides lead to inefficient phase matching within the PPLN crystal, reducing conversion efficiency [27,28]. This sensitivity presents a significant challenge when designing and implementing PPLN-based systems for broad-linewidth applications. Comprehensive studies or published reports on the design and utilization of PPLN crystals specifically for efficiently converting broad-linewidth green light to C-band spectra have yet to be published. Addressing this gap is critical for advancing optical remote sensor technologies, particularly enhancing the performance and reliability of green light optical sensor systems integrated with fiber optic networks.

This study is paramount as it demonstrates the integration of optical sensors and telecommunication technologies using PPLN. While the potential of PPLN in broad-linewidth wavelength conversion is well known, the true significance of this research lies in its innovative approach to bridging the gap between sensors and telecom systems. By employing PPLN for this purpose, the study leverages its high-efficiency conversion capabilities to handle broad-bandwidth signals effectively. Additionally, the requirement for watt-level pump power was cleverly addressed by utilizing telecom-based SHG, ensuring sufficient preparation for the conversion process. Therefore, this research confirms the robustness of PPLN in such applications and highlights a practical solution for enhancing the performance of gas sensing systems used to detect gasses like ozone, nitrogen dioxide, and volatile organic compounds in various environmental and industrial settings.

This paper presents a proposed PPLN-based integration of free-space to SMF and green to C-Band wavelength conversion for optical remote sensing applications. PPLN-based integration can convert signals from the high-turbidity water channel’s minimum loss wavelength of 518 nm to the optical fiber’s minimal loss wavelength of 1540 nm. This conversion enables seamless connectivity to standard single-mode fiber (SMF) to transmit visible spectral signals. We employ SHG using a high-power, narrow-linewidth 780 nm pump light. We then perform a wavelength conversion of a pseudo-random binary sequence (PRBS) through DFG. This study evaluates changes in the power attenuation and wavelength shift of visible light band signals input into the PPLN-based integration, assessing the resulting impact on the receivable optical signal-to-noise ratio (OSNR), defined as the optical signal-to-noise power within a 0.1 nm bandwidth, and wavelength conversion efficiency.

The remainder of this paper is organized as follows. Section 2 describes the overall configuration of the optical remote sensing system and compares the FSFC configurations. Section 3 explains the configuration for long-wavelength down-conversion. Section 4 describes the mathematical loss model of gas space. Section 5 describes the design concept of DFG from green to C-band light based on the PPLN waveguide. Section 6 and Section 7 describe the experimental setup and results for green to C-band wavelength conversion. Section 8 concludes the paper.

## 2. Optical Remote Sensing System with FSFCs

**Figure 1:**
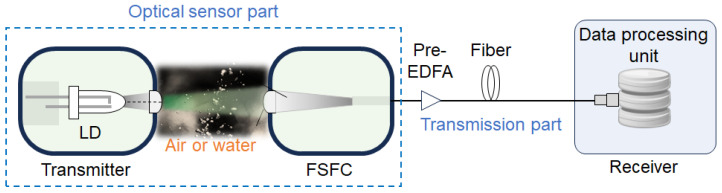
Overall structure of optical remote sensing system.

Figure 1 shows the overall configuration of an optical remote sensing system consisting of an optical sensor part and a transmission part. In the optical sensor part, light is emitted from the transmitter’s laser, passes through the air or water channel to be sensed, and is injected into the optical fiber. The FSFC, a key component in this process, ensures the efficient transmission of the sensing light. The sensing light from the FSFC is transmitted via the optical fiber to the data processing unit in the receiver, where it is received and signal-processed to obtain the sensor information. It is important to note that the sensor signal must not be significantly affected by the transmission through the optical fiber to ensure the accurate propagation of the information acquired by the optical sensor.

**Figure 2:**
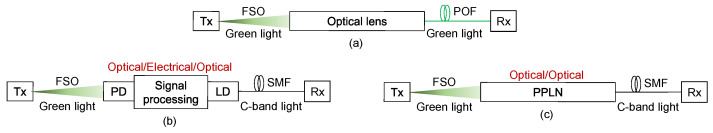
FSFC configuration: (**a**) no-conversion type, (**b**) O/E/O conversion type, and (**c**) O/O conversion type.

Figure 2a–c illustrate the configurations of three types of FSFCs: no wavelength conversion, optical/electrical/optical (O/E/O) conversion, and O/O conversion types. Table 1 summarizes the characteristics. The no-wavelength-conversion type uses plastic optical fiber (POF) to extend green light signals through wiring and is applied in indoor and underwater environments. By connecting POF, which has a large core diameter and minimal loss at 850 nm, to a passive FSFC, an extension with low loss is effective for relatively short distances compared to standalone free-space transmission. For long-distance transmission through fiber, it is preferable to use active FSFCs for wavelength conversion to the C-band, followed by SMF transmission. However, due to the band limitation, high latency, and hardware complexity of O/E/O types composed of photodetectors (PDs), signal processing, and laser diodes (LDs), the O/O type, constituted of PPLN, is desirable. When wavelength conversion is performed using an O/E/O-type optical repeater, a delay of around 100 ms occurs. Therefore, it is unsuitable for optical sensor use in next-generation wireless communication standards that require low latency.

## 3. Long-Wavelength Down-Conversion from Green Light to C-Band

**Figure 3:**
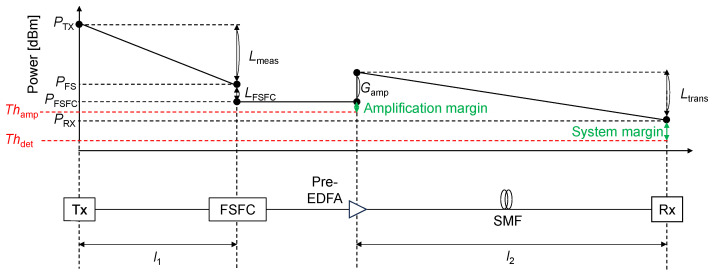
Power budget diagram of overall optical remote sensing system.

The proposed optical remote sensing system comprises a green light optical sensor and C-band optical fiber transmission, as shown in Figure 3. Additionally, a power budget diagram is also presented. The output power of the green light emitted from the transmitter is denoted as *P*_TX_. The green light segment represents the space channel between the optical transmitter and the FSFC, where the optical sensing loss during measurement is defined as *L*_meas_. The total loss from green light to C-band within the FSFC is denoted as *L*_FSFC_, and the Erbium-doped fiber amplifier (EDFA) gain is denoted as *G*_amp_. The C-band optical fiber communication segment is the SMF channel between the FSFC and the optical receiver, covering transmission loss, *L*_trans_. Let *l*_1_ be the distance from the transmitter to the FSFC and *l*_2_ be that from the pre-EDFA to the receiver. Based on these relationships, the optical power before the FSFC, *P*_FS_, the optical power after the FSFC, *P*_FSFC_, and the receiver, *P*_RX_, can be expressed as follows:

(1) $$L_{\mathrm{meas}}=P_{\mathrm{FS}}-P_{\mathrm{TX}}$$

(2) $$L_{\mathrm{FSFC}}=P_{\mathrm{FSFC}}-P_{\mathrm{FS}}$$

(3) $$L_{\mathrm{trans}}=P_{\mathrm{RX}}-P_{\mathrm{FSFC}}-G_{\mathrm{amp}}$$

As the wireless channel loss of optical sensor increases, the input power to the FSFC decreases. Consequently, the signal’s power converted to the C-band falls below the requirement threshold input power *Th*_amp_ for EDFA, making amplification unachievable. Additionally, the power difference between *P*_FSFC_ and *Th*_amp_ corresponds to the amplification margin. When *P*_RX_ is under the detection limit of the optical power meter, denoted as *Th*_det_, the optical sensing system cannot accurately measure the losses in the sensor section. Consequently, this discrepancy prevents the system from correctly correlating the losses to the sensing target, resulting in inaccurate sensor operation. Additionally, the power difference between *P*_RX_ and *Th*_det_ corresponds to the sensor system margin. Based on the minimum detectable optical power of currently commercialized optical power meters, *Th*_det_ is −70 dBm [29].

**Figure 4:**
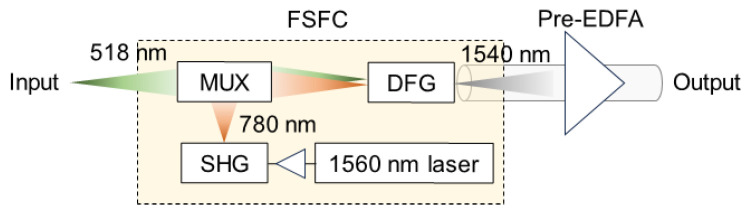
Schematic configuration of the FSFC.

Figure 4 shows a schematic configuration of the FSFC. The FSFC comprises a 1560 nm light source, an SHG, a MUX, and a DFG. In the FSFC, the output of the 1560 nm light source is input to the SHG after amplification by an EDFA. The pump light at 780 nm is then input to the MUX. After the primary signal at 518 nm is combined with the 780 nm pump light in the MUX, the signal light is converted to 1540 nm by the DFG. The pre-EDFA amplifies the wavelength-converted signal. The output of the pre-EDFA is transmitted to the receiver via SMF.

**Figure 5:**
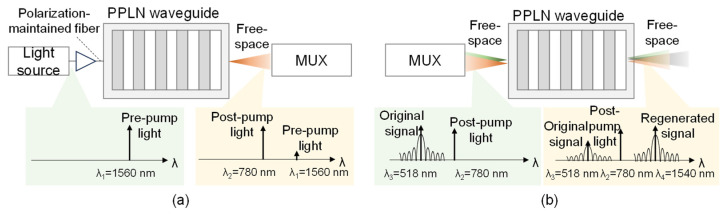
Structure of (**a**) SHG and (**b**) DFG.

Figure 5a,b show the detailed configurations of the SHG and DFG generators. The SHG is produced using a 1560 nm light in a PPLN waveguide, converting it to a 780 nm pump light. The DFG generator employs another PPLN waveguide with 518 nm signal light and 780 nm pump light to generate DFG, resulting in a 1540 nm regenerated signal light.

## 4. Mathematical Loss Model of Gas Space [30]

We consider the absorption and scattering contributions to model the loss of green light in air when detecting O_3_, NO_2_, and VOCs. The overall loss can be modeled using the Beer–Lambert law. The loss model for green light in air spacing is

(4) $$L_{\mathrm{meas}}=-\alpha l_{1}\log_{10}(e)$$

where *α* [/km] is the medium’s total attenuation coefficient in units of per kilometer, including both absorption and scattering. The attenuation coefficient *α* can be expressed as follows:

(5) $$\alpha =\alpha_{\mathrm{abs}}+\alpha_{\mathrm{scat}}$$

The absorption coefficient *α*_abs_ for each gas can be calculated based on its specific absorption cross-section *σ*_λ_ at the laser wavelength and its concentration, *C*,

(6) $$\alpha_{\mathrm{abs}}=\sigma_{\lambda ,O3}C_{O3}+\sigma_{\lambda ,\mathrm{NO}2}C_{\mathrm{NO}2}+\sigma_{\lambda ,\mathrm{VOC}}C_{\mathrm{VOC}}$$

where σ*_λ_*,_O3_, σ*_λ_*,_NO2_, and σ*_λ_*,_VOC_ are the absorption cross-sections of O_3_, NO_2_, and VOC gas at 532 nm. *C*_O3_, *C*_NO2_, and *C*_VOC_ are the concentrations of O_3_, NO_2_, and VOC gas. The scattering coefficient *α*_scat_ can be calculated using Rayleigh scattering principles:

(7) $$\alpha_{\mathrm{scat}}=\frac{24\pi^{3}{\left(n^{2}-1\right)}^{2}}{N^{2}\lambda^{4}}\left(\frac{6+3\delta }{6-7\delta }\right)P$$

where *n* is the refractive index of air in the green light wavelength. *N* is the number density of air molecules. *λ* is the wavelength of green light. *δ* is the depolarization factor. *P* is the pressure. By integrating these factors, we can predict the loss of green light due to absorption and scattering in the presence of these gasses.

## 5. Design Concept of DFG from Green to C-Band Light Based on PPLN Waveguide

We review the theory of DFG of single-mode light from an angular frequency of *ω*_g_ to *ω*_t_ using a pump light at *ω*_p_ (=*ω*_g_ − *ω*_t_), based on a second-order nonlinear optical interaction [31,32]. The model has been confirmed by many experiments to successfully explain DFG/SFG processes based on nonlinear optical interaction with a waveguide structure [20,21,22]. The subscripts g, t, and p represent green (*λ*_g_ = 518 nm), telecom (*λ*_t_ = 1540 nm), and pump light (*λ*_p_ = 780 nm), respectively. We note that, the wording used in this paper, i.e., pump, signal and converted lights, corresponding to *ω*_p_, *ω*_g_ (>*ω*_p_, *ω*_t_) and *ω*_t_, respectively, is different from the wording used in other nonlinear optical processes such as OPA, in which the strong light at the highest frequency is called the pump, the light with the seed is the signal, and the other is the idler.

We assume that the pump light is sufficiently strong and undepleted by the DFG process. When the phase-matching condition is satisfied, the photon conversion efficiency is described by

(8) $$\eta =\sin^{2}\left(\sqrt{BPl^{2}}\right)$$

where *P* is the pump power, *l* is the length of the interaction. *B* is a coupling constant described by $B=2\omega_{g}\omega_{t}d^{2}/(n_{g}n_{t}n_{p}A)$, where d represents half of the second-order nonlinear susceptibility, *n_i_* for *i = g, t, p* is the refractive index, and *A* is the cross-sectional area. For $\sqrt{BPl^{2}}=\pi /2$, *η* = 100% is obtained. From the equation, the efficiency from the input power *P*_g_ to the output power *P*_t_ is described by

(9) $$\frac{P_{t}}{P_{g}}=\frac{\omega_{t}}{\omega_{g}}\sin^{2}\left(\sqrt{BPl^{2}}\right).$$

The output power of the telecom light is proportional to the input power of the green light.

In our experiment, we use the PPLN waveguide for the second-order nonlinear optical medium to obtain high conversion efficiency using a feasible pump power. The PPLN crystal satisfies the type-0 quasi-phase-matching (QPM) condition to receive the large nonlinearity related to constant *d*, at the cost of a slightly smaller effective nonlinear susceptibility, i.e., d→2d/π, for the first-order QPM condition in our case. In the condition, only the extraordinary polarization is relevant to the DFG process. The waveguide structure allows for a long interaction time. The length of the waveguide is *l* = 20 mm, much longer than typical lengths of bulk crystals. The waveguide is a ridged type, and the cross-section of the core layer is a square with sides of 15 µm. Due to the chip design, only sub-watt-class pump power is expected to be required for maximum conversion efficiency, which differs from cases using bulk crystals. To prepare the sub-watt-class pump power at 780 nm, we use the SHG of a 1560 nm light emitted from an external-cavity diode laser after amplification by an EDFA. Using another PPLN waveguide, a sub-watt-class power of 780 nm light is obtained without the amplification after SHG. Compared with the direct emission of the 780 nm light with high energy, this method based on the PPLN waveguide and conventional telecom laser equipment will be simple, stable over the long term, and easy to maintain.

**Figure 6:**
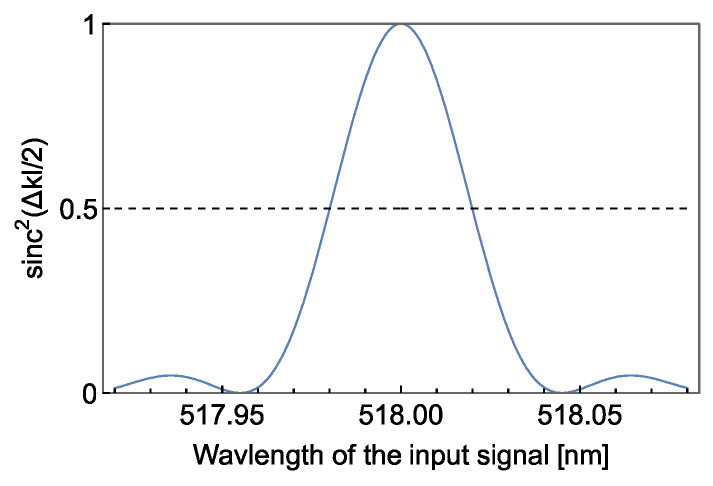
The acceptance bandwidth by sinc^2^(Δ*kl*/2) for a single-frequency pump laser at 780 nm.

The DFG of the green light by the PPLN waveguide has an acceptance bandwidth. To illustrate this, we estimate the bandwidth of QPM using the refractive indices given by the Sellmeier equation [33]. Figure 6 is a plot of sinc^2^(Δ*kl*/2) reflecting a phase matching condition, in which a single-frequency pump laser at 780 nm is assumed. Δ*k* is the phase mismatch among the three waves, and is equal to zero for the wavelengths of *λ*_g_, *λ*_t_ and *λ*_p_ at 50 °C using the poling period 6.81 μm for the simulation. From the result, the full width at half maximum bandwidth of DFG is estimated to be 44.5 GHz, which corresponds to 0.04 nm around *λ*_g_.

## 6. Experimental Setup

**Figure 7:**
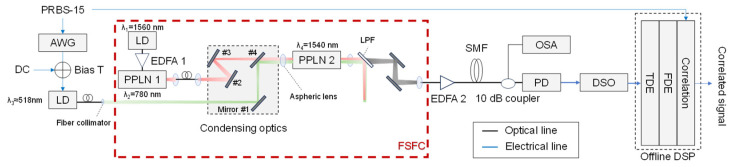
Experimental setup.

**Figure 8:**
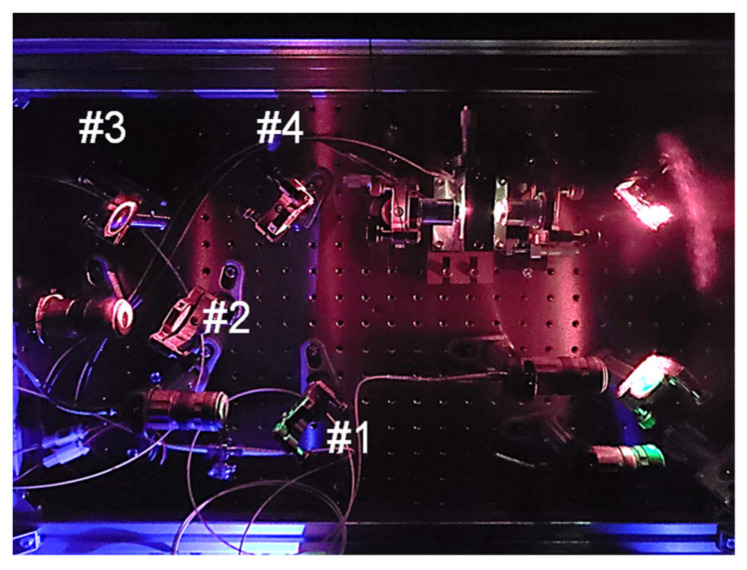
External view of FSFC.

Figure 7 displays an experimental setup for the FSFC that performs long-wavelength down-conversion from the green spectrum to the C-band. Figure 8 is a photograph showing the details of the appearance of the FSFC. In the context of sensing, considering the significant losses that occur in air or water, we utilize correlation processing in the digital domain to enhance noise tolerance through peak power detection. While correlation processing can reduce speed when transmitting data at high rates, this is not a significant issue, given that the state changes in air and water are relatively slow. Therefore, setting an appropriate correlation length corresponding to the received samples used in digital signal processing (DSP)-based correlation processing can minimize the impact on performance. The transmitter uses a 312.5 Mbps, on–off keying (OOK)-based PRBS-15 sequence. This sequence is output as an electrical signal from the arbitrary waveform generator. It has a direct current component added by a bias T. A collecting lens concentrates the directly modulated OOK signal from the LD (Thorlabs, LP520-SG15A, Newton, NJ, USA), which is input into the FSFC.

Inside the FSFC, pump light from the LD (RIO, PLANEX) with a central wavelength of 1560 nm and a linewidth of 1.64 kHz is amplified by a polarization-maintaining-type EDFA1 and then converted to 780 nm by PPLN 1 (NTT Innovative Devices, NTT Electronics Corporation, Kanagawa, Japan). The waveguide is 34 mm long, and except for the QPM condition, the chip design is similar to that of PPLN 2 (NTT Innovative Devices), described in detail in Section 5. The obtained pump light at 780 nm for DFG and the signal light at 518 nm, both of which are sent through polarization-maintaining fibers (PMFs), are combined with a dichroic mirror (#4) in the condensing optics, comprising a mirror group. The combined light is then focused on PPLN2 using an aspherical lens with a focal length of 8 mm for DFG. After PPLN2, the converted light at 1540 nm is extracted from the 518 nm signal and the 780 nm pump light passes through a long-pass filter (LPF) followed by mirrors; then, it is coupled to a SMF. The coupled light is amplified by a non-polarization-maintaining-type EDFA 2. The SMF used in the transmission path after wavelength conversion is configured using G652D fiber. It is characterized by its low attenuation and optimized performance in the 1310 nm and 1550 nm wavelength ranges. The G652D fiber also has low-polarization mode dispersion, making it suitable for high-speed optical communication. This configuration combines 1 km and 2 km bobbin fibers.

A 10 dB optical coupler separates the main and auxiliary lines on the receiver side. The main line analyzes the temporal waveform of the received signal. In contrast, the auxiliary line uses an optical spectrum analyzer to analyze the optical spectrum of the post-conversion 1540 nm signal. The PD converts the signal to an electrical signal and is sampled at 2.5 Gsample/s by a digital oscilloscope on the primary line side. The correlation signal, representing the correlation between the transmitted and received signals, is generated through time-domain and frequency-domain equalization in offline digital signal processing for correlation processing.

## 7. Experimental Results

**Figure 9:**
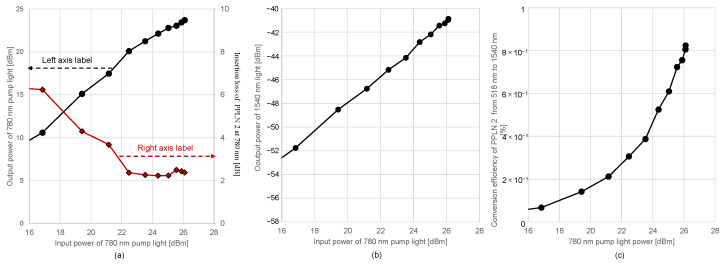
Characteristics of the change in the 780 nm pump power: (**a**) insertion loss in PPLN 2, (**b**) output power relationship, and (**c**) conversion efficiency *η* in Equation (8) (linear scale on the vertical axis).

The output power and insertion loss for the input power of the 780 nm pump light in PPLN 2 is shown in Figure 9a. The 780 nm pump power was measured before and after PPLN 2. It was observed that when the input power of the 780 nm pump light was 22.5 dBm or higher, it could be utilized efficiently with low loss. We fixed the pump power at 26.1 dBm for the remainder of the experiments. Figure 9b illustrates the relationship between the output power at 1540 nm and the change in the 780 nm pump power. The power of the 518 nm signal light was fixed at −15.31 dBm. Similarly to the changes in the 780 nm signal light power, the output power at 1540 nm varied accordingly. Figure 9c shows the efficiency of conversion *η* to the 1540 nm output light as a function of the 780 nm pump power in Equation (8). The 518 nm signal light was fixed at −15.31 dBm and input to PPLN 2. The conversion efficiency to the 1540 nm output light was found to vary with the 780 nm pump power changes.

**Figure 10:**
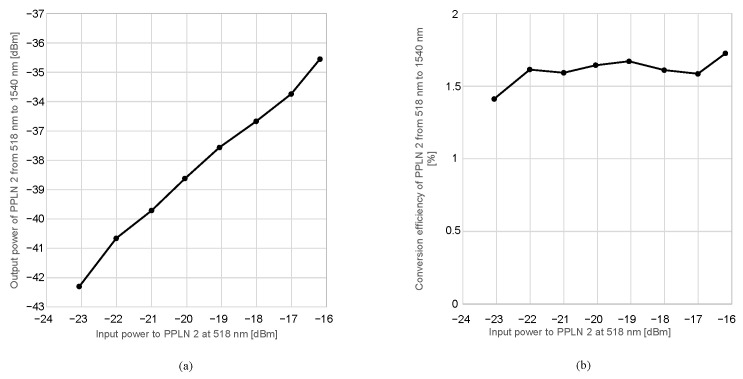
Characteristics of change in the 518 nm input power: (**a**) output power relationship and (**b**) conversion efficiency.

Figure 10a presents the relationship between the 518 nm signal light power and the 1540 nm output light power. The lower limit of the 518 nm input power in the input-output characteristics in Figure 10a is set to cover the minimum detectable optical power above −70 dBm when transmitting the wavelength-converted 1540 nm signal. The 780 nm pump light power was fixed at 26.1 dBm. As the 518 nm signal light power increased, the 1540 nm output light power was observed to increase monotonically. Figure 10b depicts the conversion efficiency in PPLN 2 for the 1540 nm output light. The output power of EDFA 1 was set to 30.79 dBm, and the output of the 780 nm pump light was fixed at 26.1 dBm. The conversion efficiency, independent of the 518 nm signal light power, varied between 1.41% and 1.7%, with an average of 1.6%.

The efficiency decreased mainly due to phase matching conditions, as the laser linewidth of 518 nm light was significantly broader at 0.5 nm compared with the predicted acceptable bandwidth of 0.04 nm, based on calculations. The laser linewidth was measured by setting the optical spectrum analyzer’s resolution to 0.1 nm and recording the spectral width at the −3 dB point from the peak value of the green laser’s optical spectrum. The effect limits the maximum conversion efficiency to 8%. The observed coupling efficiencies to the PPLN waveguide of the 518 nm light and to the SMF of the 1540 nm converted light were both approximately 50%. These effects further suppress the conversion efficiency to 2%. Consequently, considering the impact of the polarization fluctuations from the laser source, which reduce conversion efficiency by half at the maximum if the polarization is randomized, observed conversion efficiencies, such as 0.83% and 1.6% in Figure 9c and Figure 10b, are consistently explained using the experimental parameters. To improve the conversion efficiency, narrowing the laser linewidth at 518 nm is crucial. A shorter waveguide is also effective since the acceptance bandwidth is inversely proportional to the chip length, at the cost of a larger pump power. Coupling efficiencies will be improved for the optimization of the lens system used in the FSFC. Finally, the effect of the polarization fluctuations will be resolved by polarization stabilization before the PPLN 2 or by using a polarization-insensitive wavelength conversion system [21,22,34].

**Figure 11:**
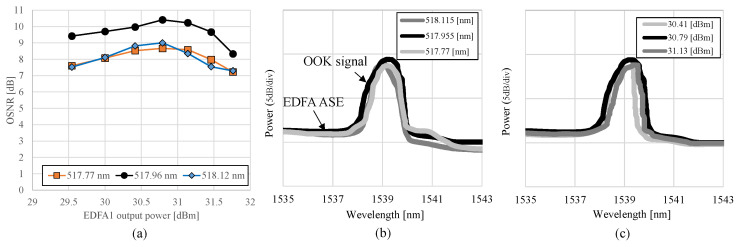
(**a**) Received OSNR characteristics in relation to the output power of EDFA 1, (**b**) optical spectrum with a varying center wavelength of the green band’s OOK signal, and (**c**) optical spectrum with a varying output power of EDFA 1.

Figure 11a displays the dependencies of OSNR characteristics on the pump power used for the wavelength conversion when the center wavelength of the continuous wave (CW) light in the green spectrum was changed. The measurement was conducted using an optical spectrum analyzer with a resolution of 0.1 nm. The OSNR is the difference between the amplified spontaneous emission noise level and the peak value of the 1540 nm light. The input light power of the green spectrum also varied during this process. The CW center wavelengths of the green spectrum were set at 517.77 nm, 517.96 nm, and 518.12 nm. The pump power was set at 26.1 dBm at each green spectrum’s center wavelength, resulting in the maximum OSNR. Figure 11b,c depict the changes in the spectral characteristics of the OOK signals in the C-band spectrum under different conditions. The resolution of the optical spectrum analyzer was set to 0.1 nm. Figure 11b shows the spectral shifts when the center wavelength of the green band OOK signal was changed, resulting in changes in the spectral peak due to wavelength adjustments. It can be observed that the 1540 nm C-band spectrum also had high power when the 517.96 nm green spectrum was incident. Figure 11c shows the spectral changes with the adjustment of the output power of EDFA 1, clarifying the impact of the wavelength conversion efficiency on the received spectrum. It can also be confirmed that when the output power of EDFA 1 was 30.79 dBm, the 1540 nm C-band spectrum had high power. The received OSNR in this experiment was affected by the broad linewidth (0.5 nm) of the 518 nm laser, which reduced the conversion efficiency of the PPLN. Narrowing the laser linewidth is expected to improve the conversion efficiency and enhance the OSNR.

**Figure 12:**
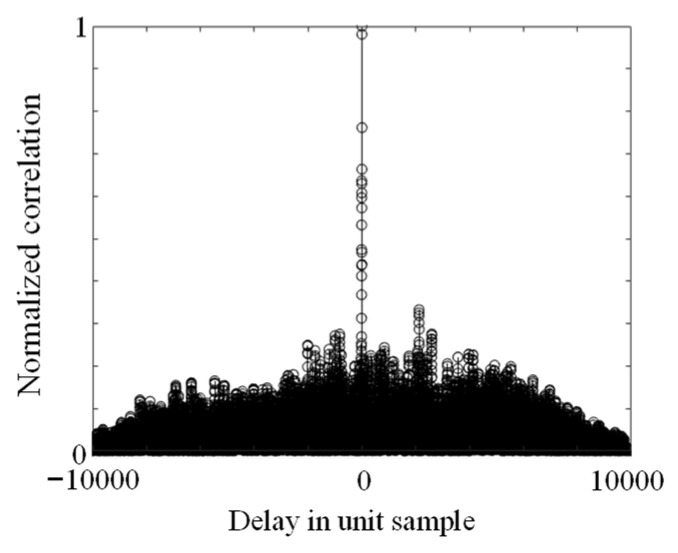
Correlation between the transmitted and received signals.

Figure 12 shows the correlation waveform of the green band OOK transmission symbols and the received symbols of the C-band OOK signal at equal sampling. The vertical axis represents a linear scale. Samples/delay is defined as the delay of the transmitted samples relative to the received samples, with 0 set as the baseline when the timing of the received and transmitted samples aligns. This correlation characteristic is obtained by normalizing the result of the correlation processing between the 2^15^ PRBS sampling used at transmission and the 20,000 sampling points equalized by the offline DSP at reception. The correlation waveform was obtained using a PD without a trans-impedance amplifier (TIA). The low OSNR, caused by the low-power EDFA input, combined with the absence of a TIA after photoelectric conversion, leads to a low electrical signal-to-noise ratio due to thermal noise from the PD. However, with a main-to-second peak ratio of 2.9 in the correlation signal, proper threshold processing enables the accurate detection of the signal components. Unlike the OSNR measurement, which directly measures the optical signal with an optical spectrum analyzer, O/E conversion introduces additional electrical noise, resulting in different measurement conditions.

**Figure 13:**
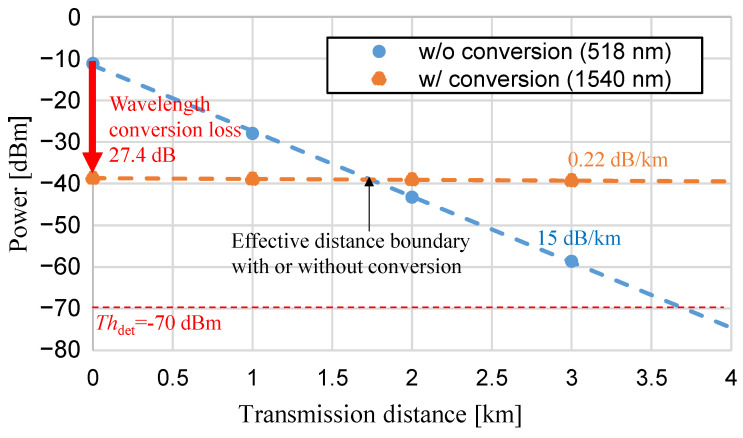
Measured power characteristics of SMF without and with wavelength conversion.

Figure 13 shows the measured power characteristics of SMF with and without wavelength conversion. For the experimental conditions, 1 km and 2 km fiber spools were used to establish three transmission distance conditions: 1 km, 2 km, and 3 km. The same SMF was utilized for the green band and the C-band. After SMF transmission, the optical power was measured using an optical power meter. The transmission loss in the green band without wavelength conversion was very high at 15 dB/km (*α* = 35/km in Equation (5)), but with wavelength conversion to the C-band, the transmission loss was low at 0.22 dB/km (*α* = 0.51/km in Equation (5)). The loss value obtained here is close to the typical value specified by ITU-T [35]. Without C-band conversion, the sensor signal reached the detection limit of the optical power meter after transmission over several kilometers of SMF, making it difficult to apply as an optical remote sensing system. On the other hand, it was confirmed that the C-band, with wavelength conversion, co be relayed through EDFA optical amplification if it does not fall below the amplification limit. The power loss at a distance of zero was 27.4 dB, with a wavelength conversion loss of 22.7 dB, corresponding to a power conversion efficiency of 1.6% × 518 nm/1540 nm = 0.54% according to Equation (9); additional experimental imperfections such as fiber joint losses of 4.7 dB were also observed in the transmission experiment. For that reason, the power without wavelength conversion was initially higher. However, after 1.7 km of propagation, the power with wavelength conversion became higher than that without wavelength conversion. Without wavelength conversion, the transmission distance to reach *Th*_det_ was 3.7 km.

## 8. Conclusions

In this study, we successfully demonstrated a proof-of-concept for an integrated optical remote sensing system capable of all-optical bulk wavelength conversion across a 1000 nm range, from green light to the C-band spectrum, transitioning from free space to fiber optics. This work addresses critical challenges in optical sensor technologies, particularly for high-precision, non-contact, and remote gas sensing applications. The integration of PPLN-based wavelength conversion within the system effectively achieved seamless connectivity between visible spectrum signals and standard SMF networks.

Our experiments showed that by carefully adjusting input parameters, such as the green light’s center wavelength, input power, and 1560 nm light power, we could achieve high OSNR after long-wavelength down-conversion for both CW light and 312.5 Mbps OOK-based PRBS signals. Notably, the transmission loss was significantly reduced from 15 dB/km in the green band to just 0.22 dB/km in the C-band, indicating a substantial improvement in transmission efficiency. The transmission limit without EDFA amplification was 3.7 km. Currently, with wavelength conversion, the power crosses with that of no wavelength conversion at 1.7 km. However, this crossing point is expected to occur at even shorter distances in the future. Therefore, wavelength conversion enables communication that significantly exceeds the transmission limit distance. Still, after wavelength conversion to the C-band, the system’s optical power levels exceeded the amplification threshold, ensuring sufficient OSNR for successful EDFA relay transmission.

These results validate the potential of PPLN-based wavelength conversion systems in enhancing the performance and reliability of green light optical sensors integrated with fiber optic technologies. Our findings suggest that this approach is particularly well suited for underwater sensing applications, where green light’s low absorption in water is advantageous.

Future research will further refine the system by applying anti-reflection coatings to optical components to minimize signal loss and enhance overall signal quality. Additionally, we will explore improvements in mode matching concerning bandwidth and polarization control to optimize conversion efficiency in PPLN crystals, particularly for input signals transitioning from free space. These advancements are expected to pave the way for next-generation optical communication systems, integrating the strengths of green light optical sensors with the robustness of fiber optic networks, ultimately enhancing remote sensing applications in environmental monitoring, industrial safety, and beyond.

## Figures and Tables

**Table 1:** Comparison of FSO/fiber network.

	No Wavelength Conversion	O/E/O Conversion	O/O Conversion
Fiber loss	High	Low	Low
Latency	Low	High	Low
Simplicity	Simple	Complicated	Simple

## Data Availability

The original contributions presented in the study are included in the article, further inquiries can be directed to the corresponding author.
